# Serial multiple mediation of the association between internet gaming disorder and suicidal ideation by insomnia and depression in adolescents in Shanghai, China

**DOI:** 10.1186/s12888-020-02870-z

**Published:** 2020-09-23

**Authors:** Yuelin Yu, Xue Yang, Suping Wang, Huwen Wang, Ruijie Chang, Lhakpa Tsamlag, Shuxian Zhang, Chen Xu, Xiaoyue Yu, Yong Cai, Joseph T. F. Lau

**Affiliations:** 1 School of Public Health, School of Medicine, Shanghai Jiao Tong University, Shanghai, 200025 PR China; 2 Centre for Health Behaviors Research, JC School of Public Health and Primary Care, the Chinese University of Hong Kong, Hong Kong, 810016 SAR China

**Keywords:** Internet gaming disorder, Insomnia, Depression, Suicidal ideation, Mediation

## Abstract

**Background:**

High prevalence and strong relationships among suicidal ideation, Internet gaming disorder (IGD), insomnia, and depression have been reported for adolescents worldwide, but the mechanism underlying these psychological problems remains unclear. This cross-sectional study explored the mediating effect of insomnia and depression on the association between IGD and suicidal ideation.

**Methods:**

Participants were 1066 adolescents (median age = 13.0 years) with Internet games exposure in the previous 12 months from junior high schools in Shanghai, China. Questionnaire measures of suicidal ideation, IGD, insomnia, depressive symptoms, and background characteristics were obtained. Path analysis was conducted to test the multiple mediating roles of insomnia and depression.

**Results:**

Suicidal ideation, IGD, insomnia, and depression prevalence was 27.2, 13.6, 9.2, and 17.0%, respectively. A serial multiple mediation model was generated. The mediation effect of insomnia and depression on the pathway from IGD to suicidal ideation was 45.5% (direct effect: standardized estimate [Std. estimate] = 0.186; total indirect effect: Std. estimate = 0.155). The association between IGD and depression was partially mediated by insomnia (direct effect: Std. estimate = 0.211; indirect effect: Std. estimate = 0.135). The proposed model fit the data well.

**Conclusions:**

Insomnia and depression may serially mediate the association between IGD and suicidal ideation. IGD was positively associated with insomnia, then with depression, which in turn positively contributed to suicidal ideation. We suggest greater monitoring of Internet use and prevention of insomnia and depression to mitigate the risk of suicidal ideation among Chinese adolescents.

## Background

Adolescence is one of the life stages that exhibits higher prevalence of suicides [1] and having suicide as the second leading cause of death (U.S. CDC). Suicidal ideation predicts subsequent suicidal plans, attempts, and behaviors [2, 3]. The global 12-month and lifetime prevalence for adolescent suicidal ideation was 14.2 and 18%, respectively [4]. The prevalence of suicidal ideation has increased in Chinese adolescents. For instance, it increased from 10.4 to 13.6% among grade 8–9 Hong Kong Chinese students according to a 1-year longitudinal study [5]. Given the rising prevalence and harmful consequences of suicidal ideation, as well as a tendency for suicidal ideation to appear at younger ages [6], early identification and effective intervention are warranted to reduce adolescent suicidal ideation and its risk factors.

Recent research on suicidal ideation has investigated adolescents with Internet gaming disorder (IGD). IGD is a another worldwide public health problem and has been included in ICD-11 in 2018 [7]. The overall global prevalence of adolescent IGD was 6.0% [8], and was highest in east Asian countries [9]. IGD was associated with psychological disorders such as insomnia [10, 11], depressive symptoms [12, 13], loneliness, anxiety [14], and suicidal ideation [15–17]. A body of studies have shown that people addicted to internet would have a higher risk of suicidal ideation [18]. A dose–effect relationship was found between Internet game exposure of ≥5 h per day and suicidal ideation (odds ratio [OR] = 1.7; 95% confidence interval [CI]: 1.3–2.1) [19]. Yet, few studies have explored the psychological factors that lead from IGD to suicidal ideation, and the mechanism underlying this association remains unclear.

The substantial association between both IGD and suicidal ideation and psychiatric problems such as insomnia and depression may shed insights to our understanding about the mechanism. Depression is one of the strongest predictors of suicidal ideation [20]. Majority (> 90%) of people who committed suicide express depressive symptoms simultaneously, and this co-occurrence rate in adolescent suicide cases is more than 50% [21]. Similarly, adolescents with insomnia problems are more likely to generate suicidal ideation. One recent meta-analysis identified insomnia as a predictor of suicidal ideation (OR = 2.35; 95% CI: 1.58–1.92) [22]. Systematic reviews have also shown that insomnia (OR = 2.20; 95% CI: 1.77–2.74) [23] and depression (OR = 1.22; 95% CI: 1.10–1.34) [24] are among the strongest correlates of IGD. A recent study indicates that depression mediates the association between addiction to mobile phone use and suicidality in Chinese adolescents (95% CI: 27.891–69.831) [25]. Given their strong association with both IGD and suicidal ideation, insomnia and depression may be key mediators in the relationship between IGD and suicidal ideation. If so, this would help to narrow the causal gap between IGD and suicidal ideation.

Previous studies have suggested that insomnia can potentially mediate the relationship between IGD and depression, indicating that insomnia and depression may sequentially mediate the effect of IGD on suicidal ideation among adolescents. One meta-analysis of thirty-four cohort studies have revealed that insomnia serves as a strong predictor on depression [26]. There is a bidirectional causal relationship between IGD and insomnia; however, insomnia accounts for a greater proportion of the total effect on depression [27–29]. Thus, insomnia is accepted as a mediator on the relationship between IGD and depression. As there were strong associations as well as strong predictor effects between IGD, insomnia, depression and suicidal ideation, the following mediation model may be implied that adolescents with IGD may experience insomnia before developing depressive symptoms and, consequently, develop a high risk of suicidal ideation.

Although research suggests separate robust associations among psychological disorders, theoretical models are needed to explain how IGD affects suicidal ideation. Therefore, this study investigated a serial multiple mediation model based on previous study findings. The aim was [1] to investigate the prevalence for suicidal ideation, IGD, insomnia, and depression among adolescents in junior high school in Shanghai, China [2]; to test the association between suicidal ideation, IGD, insomnia, and depression [3]; to explore the serial multiple mediation effect of insomnia and depression on the association between IGD and suicidal ideation.

## Method

### Participants and recruitment

Using convenience sampling, six junior high schools from two districts of Shanghai, China, were selected from April to May 2018. All first-grade students (a total of 1329) from the selected schools were recruited. After removing observations with over 5% of items missing and low credibility, 1243 observations remained. Data from 1066 (85.8%) students who self-reported themselves as Internet gamers in the past 12 months were used for statistical analysis.

Permission for this in-school survey was obtained before the investigation from schools, legal guardians, and students. As school principals are responsible for students, these were first informed about the study and their consent obtained. Then students and their legal guardians were informed about the study aims and procedure. Verbal informed consent was obtained from legal guardians and students themselves as they participated in the investigation during school time. Participants were informed that all data collected would be used only for research purposes and would be strictly confidential. The background, aim, procedure, and confidentiality of the study were explained at the top of the questionnaire. Participants were free to terminate their participation at any time with no adverse consequences. All eligible participants were asked to complete an anonymous structured questionnaire in class.

### Measures

#### Background characteristics

The following background characteristics were analyzed: sex, age, mother’s educational level, father’s educational level, perceived family financial condition, residence identity (local or migrant residents), family type (single-parent family or not), and living arrangements (lives with parents or not). These background characteristics were selected by referring to the literature.

#### Suicidal ideation

Participants were asked how often over the last 12 months they had considered suicide. The three possible response options reflected the frequency of emerging suicidal ideation: “0” (never), “1” (once or twice), and “2” (more than twice). We categorized respondents into two groups for descriptive statistical analysis and logistic regression. Participants who chose “1” or “2” were deemed as exhibiting suicidal ideation and those who chose “0” were considered to show no suicidal ideation. Suicidal ideation category scale scores were used for path analysis.

#### Internet gaming disorder (IGD)

IGD was assessed using the diagnostic criteria in the Diagnostic and Statistical Manual of Mental Disorders Fifth Edition (DSM-V) [30]. The measure comprised nine items that assessed IGD symptoms. Participants rated how often they had experienced the symptoms in the previous 12 months on a yes/no scale; “0” indicated absence of the symptom and “1” indicated its presence. Positive responses on ≥5 criteria were considered to indicate IGD (Cronbach’s α = 0.746). IGD continuous scale scores were used for path analysis.

#### Insomnia

Insomnia was assessed using the Insomnia Severity Index (ISI) [31]. The ISI is a 7-item self-report instrument that measures symptoms and insomnia-related problems. The scale has been validated and is widely used in insomnia studies [32–34]. The total ISI score ranges from 0 to 28; higher scores indicate more severe insomnia. Scores of 0–7 indicate no insomnia, 8–14 indicate subclinical insomnia, 15–21 indicate moderate clinical insomnia, and 22–28 indicate severe clinical insomnia (Cronbach’s α = 0.838). Participants with a total score > 14 are deemed to have clinical insomnia [35]. ISI continuous scale scores were used for path analysis.

#### Depression

The 9-item Patient Health Questionnaire (PHQ-9) [36] was used to evaluate depression, as many previous studies indicate its effectiveness and superiority for assessing depression [37, 38]. Total PHQ-9 scores range from 0 to 27; higher scores indicate more severe depression. Scores of 0–4 indicate no depression, 5–9 indicate mild depression, 10–14 indicate moderate depression, 15–19 indicate moderately severe depression, and 20–27 indicate severe depression (Cronbach’s α = 0.870). Total scores ≥10 are considered to indicate depression [39]. PHQ-9 continuous scale scores were used for path analysis.

### Statistical analysis

Descriptive analyses were first conducted of background characteristics and the prevalence of suicidal ideation, IGD, insomnia, and depression. As the distribution of age was skewed, this continuous variable was described using the median (interquartile range [IQR]), and the median was used to divide this variable into two categories for the subsequent logistic regression. Categorical variables (suicidal ideation, IGD, insomnia level, depression level, sex, father’s educational level, mother’s educational level, perceived family financial condition, residence identity, family type, and living arrangements) were described using frequencies (percentages).

Univariate logistic regression was then performed to examine the association between background characteristics and suicidal ideation, and the association between psychological variables (IGD, insomnia, and depression) and suicidal ideation. After controlling statistically significant background characteristics, we included IGD, insomnia, and depression into a logistic regression model to obtain adjusted ORs (AORs) and the corresponding CIs. Moreover, pairwise correlation analysis of measurements (DSM-V for IGD, ISI for insomnia, and PHQ-9 for depression, questionnaire for suicidal ideation) was used to test the relationships among the variables.

The serial multiple mediation hypothesis for IGD, insomnia, depression, and suicidal ideation was tested using Preacher and Hayes’s method [40]. Bootstrapping analysis with 5000 resamples was conducted to test the significance of the mediation effects [41]. The weighted least squares and mean and variance estimator was used as the outcome was categorical. The significant background variable of suicidal ideation reported in the regression analysis was controlled. Model fit indices (root mean square error of approximation [RMSEA], comparative fit index [CFI], Tucker–Lewis index [TLI], standardized root mean square residual [SRMR]) were calculated to assess the model goodness of fit. RMSEA and SRMR values < 0.08, and CFI and TLI values > 0.90, indicated acceptable goodness of fit [42].

We used IBM SPSS Statistics 24.0 (IBM Corp., Armonk, NY, USA) to conduct the descriptive analysis, logistic regression, and pairwise correlation analysis, and used Mplus Version 8.3 (Muthen & Muthen, Los Angeles, CA, USA) to conduct the path analysis. *P* values < 0.05 were considered statistically significant.

## Results

### Descriptive analyses

As shown in Tables 1 and 2, 1,066 participants (median age [IQR] = 13.0 [12.0, 13.0] years) had been exposed to Internet games in the previous 12 months; 43.5% were female. Most participants’ parents (83.3% mother; 85.8% father) had at least senior high school education. Over 90% of participants perceived their family financial condition as above or equivalent to medium, and 11.4% of participants were living in single-parent families.

**Table 1:** Descriptive and univariate logistic regression analysis of background characteristics on suicidal ideation (≥once)

Characteristics	Number of participants	Suicidal Ideation(≥once)	
		n(row)	ORu (95% CI)
**Sex (%)**			
Male	602 (56.5%)	121 (20.1%)	Ref
Female	464 (43.5%)	169 (36.4%)	2.277 (1.730,2.997) ***
**Age (years), median (IQR)**	13.0 (12.0,13.0)		
< = 12 years (%)	342 (32.1%)	107 (31.3%)	Ref
> = 13 years (%)	724 (67.9%)	183 (25.3%)	0.743 (0.560,0.986) *
**Mother’s education level (%)**			
Primary school or below	31 (2.9%)	13 (41.9%)	Ref
Junior high school	124 (11.7%)	45 (36.3%)	0.789 (0.354,1.758)
Senior high school	219 (20.7%)	59 (26.9%)	0.511 (0.236,1.106)
University or above	664 (62.6%)	162 (24.4%)	0.447 (0.214,0.932) *
Unknown	22 (2.1%)	10 (45.5%)	1.154 (0.384,3.471)
**Father’s education level (%)**			
Primary school or below	25 (2.4%)	10 (40.0%)	Ref
Junior high school	112 (9.6%)	39 (38.2%)	0.929 (0.380,2.271)
Senior high school	222 (20.9%)	68 (30.6%)	0.662 (0.283,1.549)
University or above	689 (64.9%)	162 (23.5%)	0.461 (0.203,1.046)
Unknown	23 (2.2%)	10 (43.5%)	1.154 (0.366,3.640)
**Perceived family financial condition (%)**			
Very good/good	655 (61.5%)	144 (22.0%)	Ref
Medium	318 (29.9%)	106 (33.3%)	1.774 (1.318,2.389) ***
Very poor/poor	26 (2.4%)	16 (61.5%)	5.678 (2.522,12.781) ***
Unknown	66 (6.2%)	24 (36.4%)	2.028 (1.188,3.461) **
**Residence identity (%)**			
Local	881 (82.8%)	247 (28.0%)	Ref
Migrant	183 (17.2%)	42 (23.0%)	0.765 (0.526,1.112)
**Family type (%)**			
Non single-parent family	941 (88.6%)	249 (26.5%)	Ref
Single-parent family	121 (11.4%)	41 (33.9%)	1.424 (0.952,2.132)
**Living arrangements (%)**			
Live with parents	906 (85.2%)	237 (26.2%)	Ref
Only live with mother	77 (7.2%)	25 (32.5%)	1.357 (0.824,2.236)
Only live with father	37 (3.5%)	13 (35.1%)	1.529 (0.766,3.052)
Live with neither	43 (4.0%)	14 (32.6%)	1.363 (0.708,2.623)
**Suicidal ideation (%)**			
Never think about it	776 (72.8%)	–	
Think it more than once	290 (27.2%)	–	

*IQR* Interquartile range, *ORu* Univariate odds ratio, *CI* Confidence interval

*p < 0.05, **p < 0.01, ∗∗∗*p* < 0.001

**Table 2:** Descriptive and logistic regression analysis of psychological variables on suicidal ideation (≥once)

Characteristics	Number of participants	Suicidal Ideation(≥once)		
		n(row%)	ORu (95% CI)	AOR (95% CI)
**Internet gaming disorder (%)**				
No	921 (86.4%)	222 (24.1%)	Ref	Ref
Yes	145 (13.6%)	68 (46.9%)	2.781 (1.941,3.983) ***	3.089 (2.100,4.544) ***
**Insomnia condition (%)**				
None insomnia (0–7)	663 (62.2%)	113 (17.0%)	Ref	Ref
Subclinical insomnia [8–14]	305 (28.6%)	122 (40.0%)	3.245 (2.390,4.405) ***	3.052 (2.224,4.189) ***
Clinical insomnia [14–28]	98 (9.2%)	55 (56.1%)	6.226 (3.980,9.738) ***	5.751 (3.614,9.152) ***
**Depression (%)**				
None depression (0–4)	556 (52.2%)	58 (10.4%)	Ref	Ref
Mild depression [5–9]	329 (30.9%)	112 (34.0%)	4.432 (3.108,6.320) ***	4.289 (2.985,6.162) ***
Presence of depression [10–27]	181 (17.0%)	120 (66.3%)	16.891 (11.198,25.477) ***	14.805 (9.685,22.630) ***

*ORu* Univariate odds ratio, *AOR* Adjusted odds ratio for background characteristics included in this study

*CI* Confidence interval

*p < 0.05, **p < 0.01, ∗∗∗*p* < 0.001

Of adolescent Internet game players, the prevalence of suicidal ideation, IGD, clinical insomnia, and depression was 27.2, 13.6, 9.2, and 17.0%, respectively. Participants with more severe levels of psychological disorder had greater suicidal ideation. About half of adolescents with IGD (46.9%), clinical insomnia (56.1%), and depression (66.3%) showed suicidal ideation.

### Logistic regression analysis

The results of the univariate and adjusted logistic regression analysis of suicidal ideation are also shown in Tables 1 and 2. Of the background characteristics, sex (ORu = 2.277; 95% CI: 1.730–2.997), age (≥13 years: ORu = 0.743; 95% CI: 0.560–0.986), and perceived family financial condition (medium: ORu = 1.774; 95% CI: 1.318–2.389; very poor/poor: ORu = 5.678; 95% CI: 2.522–12.781) were significantly associated with suicidal ideation (Table 1).

The adjusted regression results showed that IGD (AOR = 3.089; 95% CI: 2.100–4.544), clinical insomnia (AOR = 5.751; 95% CI: 3.614–9.152), and presence of depression (AOR = 14.805; 95% CI: 9.685–22.630) were all positively associated with suicidal ideation (*p* < 0.001) (Table 2).

### Pairwise correlation analysis

Table 3 shows the results of the pairwise correlation analysis. There were positive and significant correlations among IGD, insomnia, depression, and suicidal ideation (*p* < 0.001).

**Table 3:** Questionnaire scores and pairwise correlation analysis (*N* = 1066)

	IGD scale score	ISI scale score	PHQ-9 scale score	Suicidal ideation
**IGD**	1			
**ISI scale score**	0.240***	1		
**PHQ-9 scale score**	0.338***	0.630***	1	
**Suicidal ideation**	0.273***	0.343***	0.509***	1

*IGD* Internet gaming disorder, *ISI* Insomnia Severity Index, *PHQ-9* 9-item Patient Health Questionnaire

*p* < 0.001

### Path analysis

The proposed mediation model showed an acceptable goodness of fit (CFI = 0.974; TLI = 0.901; RMSEA = 0.076; SRMR = 0.054).

**Fig. 1:**
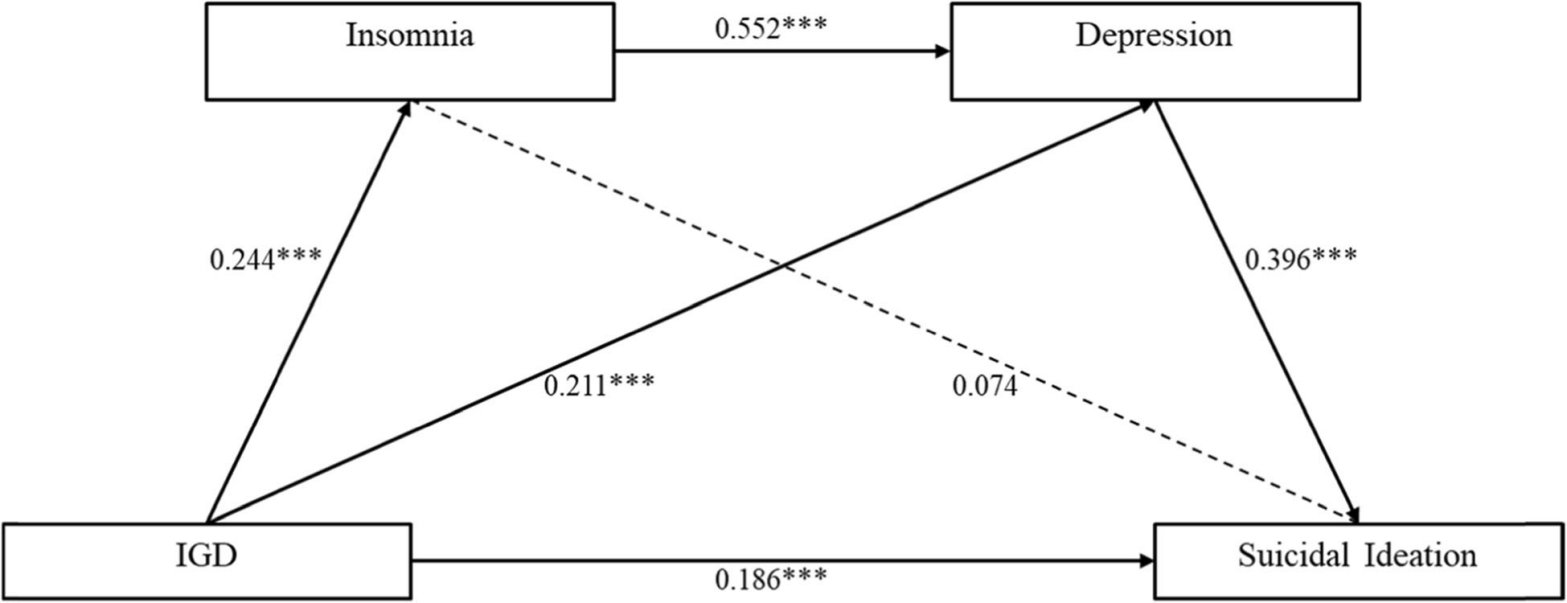
Path analysis of IGD, insomnia, depression, and suicidal ideation among adolescents (*N* = 1066) Note: IGD: Internet gaming disorder. Sex, age, and perceived family financial condition were significant in the regression analysis and were adjusted as covariates in the path analysis. Parameters displayed are standardized estimates of the direct effect on each pathway. ***p < 0.001

As shown in Table 4 and Fig. 1, there was a significant total effect (standardized estimate [Std. estimate] = 0.341, *p* < 0.001) of IGD on suicidal ideation. The direct effect of IGD on suicidal ideation was also significant (Std. estimate = 0.186, *p* < 0.001). In addition, the indirect effects of IGD on suicidal ideation through depression (Std. estimate = 0.083, *p* < 0.001) and through insomnia then depression (Std. estimate = 0.053, *p* < 0.001) were significant. However, the indirect effect through insomnia was not significant (*p* = 0.065). Overall, the mediating effect of insomnia and depression were 45.5% (0.155/0.341 [Std. estimate of total indirect effect/Std. estimate of total effect]) in the pathway from IGD to suicidal ideation. Moreover, the mediating effect of insomnia accounted for 39.0% (0.135/0.346 [Std. estimate of indirect effect/Std. estimate of total effect]) of the association between IGD and depression.

**Table 4:** Results of path analysis (*N* = 1066)

Paths	Std. Estimate	Estimate	Bootstrapping 95% CI		S.E.	Est./S.E.	***P*** value
			Lower 2.5%	Upper 2.5%			
***Depression***							
**Effect**							
**Total effect**	0.346	0.850	0.688	1.018	0.085	10.012	< 0.001
**Direct effect**							
IGD → Depression	0.211	0.518	0.382	0.658	0.071	7.268	< 0.001
**Indirect effect**							
IGD → Insomnia → Depression	0.135	0.332	0.249	0.423	0.044	7.476	< 0.001
***Suicidal Ideation***							
**Effect**							
**Total effect**	0.341	0.187	0.145	0.227	0.021	9.044	< 0.001
**Direct effect**							
IGD → Suicidal ideation	0.186	0.102	0.062	0.141	0.020	5.061	< 0.001
**Indirect effect**							
**Total indirect effect**	0.155	0.085	0.065	0.105	0.010	8.130	< 0.001
**Specific indirect effect**							
IGD → Insomnia → Suicidal ideation	0.018	0.010	0.000	0.021	0.005	1.849	0.065
IGD → Depression → Suicidal ideation	0.083	0.046	0.032	0.062	0.008	5.846	< 0.001
IGD → Insomnia → Depression → Suicidal ideation	0.053	0.029	0.020	0.040	0.005	5.927	< 0.001
***Coefficient***							
IGD → Insomnia	0.244	0.611	0.461	0.761	0.077	7.919	< 0.001
Insomnia → Depression	0.552	0.543	0.482	0.604	0.031	17.771	< 0.001
Depression → Suicidal ideation	0.396	0.088	0.071	0.104	0.009	10.148	< 0.001
***Insomnia → Suicidal ideation***							
**Effect**							
**Total effect**	0.293	0.064	0.049	0.078	0.007	8.936	< 0.001
**Direct effect**	0.074	0.016	0.000	0.033	0.008	1.919	0.055
**Indirect effect**							
Insomnia → Depression → Suicidal ideation	0.219	0.048	0.038	0.058	0.005	9.045	< 0.001

*IGD* Internet gaming disorder

*Std. Estimate (Est.)* Standardized estimate, *S.E* Standard error, *CI* Confidence interval

*p* < 0.05 was considered significant

## Discussion

This study not only provides regional prevalence data for suicidal ideation, IGD, insomnia, and depression among adolescent Internet gamers in Shanghai, China, but also elucidates the relationships and the underlying mechanisms of these four psychological disorders using a serial multiple mediation model. The prevalence for suicidal ideation in our general study samples and the specific IGD group was 27.2 and 46.9%, respectively. Compared with adolescents with normal Internet use, individuals with IGD were more vulnerable to suicidal ideation. These results accord with those of a previous study of 9758 students from five European countries [43], which found that 45.86% of students with problematic Internet gaming behaviors showed suicidality. Adolescents with other types of Internet addiction also show a substantial risk of suicidal ideation [18]. Female, younger, poorer students in our study showed a greater likelihood of suicidal ideation. These sociodemographic differences echo other findings from different countries [44, 45]. One study reported that participants with a median age of 13 years (IQR: 8–15 years) [6] showed harmful consequences of suicidal ideation and suicidal attempts that required clinical treatment.

The present findings identified significant relationships among IGD, insomnia, depression, and suicidal ideation, and strongly suggest that more efforts are needed to monitor Internet use and prevent adolescent insomnia and depression to mitigate the risk of suicidal ideation. Our results show a strong mediating effect (39.0%) of insomnia on the association between IGD and depression. This effect is similar to that found in studies conducted with Hong Kong adolescents [46] and adolescents in Nepal [29], yet much lower than the effect (60.6%) shown for adolescents in Guangzhou, China [27]. This large difference may partly reflect the study population. Although both studies targeted secondary school students, the Guangzhou study included all grades of compulsive education, whereas our study examined only the first grade. The former population may have been exposed to greater sleep deprivation owing to the stress of competing for entrance to senior high schools. Barley et al. [47] emphasized that stress is an important influence on the comorbidity of Internet addiction and insomnia. Thus, the relationships among addiction to types of Internet usage other than IGD, and insomnia and depression need to be further explored while considering the influence of different population background characteristics. Besides, our model tested the bidirectional relationship between IGD and insomnia with a statistically insignificant result, and founded that insomnia was a stronger mediator than IGD in the associations among IGD, insomnia and depression, which was in accordance with previous literature.

This is the first study to examine a serial multiple mediation model of the associations between IGD, insomnia, depression, and suicidal ideation in Chinese adolescents. The mediation model demonstrated that IGD was sequentially correlated with insomnia in the first step, and further positively affected the onset of depression, which was associated with a greater risk of suicidal ideation. Additionally, insomnia alone failed to significantly mediate the pathway to suicidal ideation, which highlights the role of depression as a key mediator in the whole model. This finding is in accordance with a study by Sami et al. [48] on the correlation between sleep disturbance and suicidal ideation. It is possible that depression is the strongest risk factor for suicidal ideation in the presence of IGD, insomnia, or other psychological disorders. It is hard to predict suicidal ideation, but the risk factors identified in this study can be measured. Therefore, better treatment for IGD, insomnia, and depression is central to the prevention of suicidal ideation in the Internet era.

This study had some limitations. First, to reduce the questionnaire length and response time, the suicidal ideation measure was a single question with three response options, which is inadequate for accurate diagnoses. A validated theory-based scale is preferable for identifying suicidal ideation in adolescents. Although many other studies [49, 50] have also used a single question (i.e., the ninth item on the PHQ-9 scale, “thoughts that you would be better off dead or of hurting yourself in some way”) to identify suicidality, which was similar to the question used here, a more comprehensive inquiry about suicidal ideation would increase the reliability of outcomes. Second, this was a cross-sectional study, and so the reliability of the findings may be low. Longitudinal studies are needed to confirm a temporal effect of these associations. Third, convenience sampling was used to obtain the study population, so the prevalence of suicidal ideation shown here may not apply to adolescents in other regions, as regional differences may affect prevalence. Finally, in addition to the two mediators tested here, other factors may be important in the pathway from IGD to suicidal ideation. For example, impulsivity is a characteristic trait of adolescence and is strongly associated with addictive disorders (e.g., IGD) and other risky behaviors (e.g., suicidal ideation) [51]. As adolescence is characterized by internal psychological change and external interpersonal adaption, future studies should examine other psychological factors that may be associated with suicidal ideation.

## Conclusions

This study is the first to explore the relationships among suicidal ideation, IGD, insomnia, and depression in Chinese adolescents. We found a serial multiple mediation effect of insomnia and depression on the pathway from IGD to suicidal ideation. Insomnia first played a partial mediating role in the association between IGD and depression, then depression in turn fully mediated the pathway from insomnia to suicidal ideation. We recommend that interventions for IGD, insomnia, and depression should be strengthened to prevent suicide among adolescents in China.

## Data Availability

Data and materials used in this study are available from the corresponding authors and will be made available on reasonable request.

## Abbreviations

IGD: Internet gaming disorder
ISI: Insomnia Severity Index
PHQ-9: 9-item Patient Health Questionnaire
IQR: Interquartile range
Std. estimate: Standardized estimate
S.E.: Standard error
ORu: Univariate odds ratio
AOR: Adjusted odds ratio
CI: Confidence interval
